# We are bitter, but we are better off: case study of the implementation of an electronic health record system into a mental health hospital in England

**DOI:** 10.1186/1472-6963-12-484

**Published:** 2012-12-31

**Authors:** Amirhossein Takian, Aziz Sheikh, Nicholas Barber

**Affiliations:** 1 Division of Health Studies, School of Health Sciences & Social Care, Brunel University London, Uxbridge, UB8 3PH, UK; 2 eHealth Research Group, Centre for Population Health Sciences, The University of Edinburgh, Edinburgh, EH8 9DX, UK; 3 Department of Practice and Policy, UCL School of Pharmacy, London, WC1H 9JP, UK

**Keywords:** Electronic health records (EHR), Mental health, ‘Sociotechnical changing’, Implementation, Adoption

## Abstract

**Background:**

In contrast to the acute hospital sector, there have been relatively few implementations of integrated electronic health record (EHR) systems into specialist mental health settings. The National Programme for Information Technology (NPfIT) in England was the most expensive IT-based transformation of public services ever undertaken, which aimed amongst other things, to implement integrated EHR systems into mental health hospitals. This paper describes the arrival, the process of implementation, stakeholders’ experiences and the local consequences of the implementation of an EHR system into a mental health hospital.

**Methods:**

Longitudinal, real-time, case study-based evaluation of the implementation and adoption of an EHR software (RiO) into an English mental health hospital known here as Beta. We conducted 48 in-depth interviews with a wide range of internal and external stakeholders, undertook 26 hours of on-site observations, and obtained 65 sets of relevant documents from various types relating to Beta. Analysis was both inductive and deductive, the latter being informed by the ‘sociotechnical changing’ theoretical framework.

**Results:**

Many interviewees perceived the implementation of the EHR system as challenging and cumbersome. During the early stages of the implementation, some clinicians felt that using the software was time-consuming leading to the conclusion that the EHR was not fit for purpose. Most interviewees considered the chain of deployment of the EHR–which was imposed by NPfIT–as bureaucratic and obstructive, which restricted customization and as a result limited adoption and use. The low IT literacy among users at Beta was a further barrier to the implementation of the EHR. This along with inadequate training in using the EHR software led to resistance to the significant cultural and work environment changes initiated by EHR. Despite the many challenges, Beta achieved some early positive results. These included: the ability to check progress notes and monitor staff activities; improving quality of care as a result of real-time, more accurate and shared patient records across the hospital; and potentially improving the safety of care through increasing the legibility of the clinical record.

**Conclusions:**

Notwithstanding what was seen as a turbulent, painful and troublesome implementation of the EHR system, Beta achieved some early clinical and managerial benefits from implementing EHRs. The ‘sociotechnical changing’ framework helped us go beyond the dichotomy of success versus failure, when conducting the evaluation and interpreting findings. Given the scope for continued development, there are good reasons, we argue, to scale up the intake of EHR systems by mental health care settings. Software customization and appropriate support are essential to work EHR out in such organizations.

## Background

Provision of mental health services often involves professionals located in disparate locations. It has been suggested that the use of integrated electronic health record (EHR) systems (a digital longitudinal record of a citizen’s health and healthcare interventions that can be accessed by healthcare providers from across a defined range of healthcare settings) will help to improve the quality of care for mental health patients [1,2] through, for example, preventing loss of records, increasing accessibility of the records, improving medication management, reducing medical errors and costs [3-5], and empowering patients through greater engagement in their care provision [6].

Despite these anticipated benefits, few implementations of integrated EHR systems have taken place across mental health settings [7,8]. Possible reasons for this lack of progress include: sensitivities in relation to the potential for stigma and discrimination associated with the unambiguous recording of diagnoses in medical records [9]; healthcare professionals’ reluctance to use EHRs [10]; concerns about lowering productivity and inaccurate clinical notes [11]; data security and confidentiality due to very sensitive and specific nature of mental health [12,13]; concerns about the quality of patient-provider relationship [14,15]; and considerably lower frequency of self-determination in decisions about mental health, compared to acute settings [16]. As a result, little has been published on the evaluation of the implementation of EHRs in the context of mental health [8].

Launched in 2002 and officially dismantled in 2011 [17,18], the National Programme for Information Technology (NPfIT) included the first sustained national attempt to introduce centrally-procured EHR systems across the National Health Service’s (NHS) hospitals [19,20], including mental health settings [21-23]. We conducted the first national evaluation of implementation and adoption of EHR systems in NHS ‘early adopter’ hospitals and have reported on this in detail elsewhere [24,25]. Here, we report on a case study of the implementation of an EHR (RiO) into a mental health setting delivered though the NPfIT and analyzed using our adapted ‘sociotechnical changing framework’ (see below for more elaboration). We investigated the arrival and the implementation process, stakeholders’ experiences and perceptions, and local consequences of adopting this nationally procured ‘off the shelf’ EHR software that, in various forms, had been used for up to 15 years in a few mental health and community centres in the UK.

## Methods

### Ethical considerations

Our research was classified as a service evaluation (ref. 08/H0703/112). We obtained informed consent from the participating hospital and individuals and guaranteed their anonymity.

### Design, sampling and data collection

This was a prospective, longitudinal [26], sociotechnical [27], and real-time case study-based evaluation [28]. Beta was selected from a purposive sample [29] of 12 diverse, NHS ‘early adopter’ hospitals studied over a 30-month period from September 2008 until February 2011. This paper focuses in-depth on one of these sites (Beta), in which the lead researcher (AT) collected a broad range of qualitative data (see Table 1 for characteristics of Beta and the dataset). We conceptualized Beta as an independent case study to reflect the importance of local contingencies [30]. This allowed the specific character of the implementation and adoption of the EHR software to be revealed, whilst attempting to make general inferences transferable to other contexts [31,32].

**Table 1:** Characteristics of Beta and the dataset

**EHR application**	**Hospital characteristics**	**Time of data collection & dataset**
RiO version 5.0;	Large;	May 2009-November 2010;
Software developer:	Multisite;	*48 face to face interviews:*
CSE Healthcare Systems	Teaching;	6 Senior manager and members of the Board
(http://www.cse-healthcare.com)	Foundation (i.e. more autonomous); covering over 500,000 people; with an annual turnover in excess of of £130m	14 Implementation team and IT managers,
		20 Healthcare practitioners: 2 inpatient nurse,
		7 community nurse, 5 psychiatric consultants & 1 junior doctor, 1 pharmacists, 3 social worker,
		1 occupational therapist, 2 Administration staff,
		4 NPfIT, 1 BT, 1 External IM & T consultant.
		*65 categories of hospital documents from various types*; and
		*26 hours on-site observation*

Within Beta, we purposefully (and at times opportunistically) conducted semi-structured interviews with a diverse range of stakeholders with broad range of perspectives, from inside and outside of the hospitals (see Table 1 for more details). We developed generic interview guides that were then tailored for specific participants [See Additional files 1, 2 and 3]. The majority of interviews were audio-recorded and transcribed verbatim. Interviews were complemented by the researcher’s field notes, as well as observational and documentary data of various types with regard to planning, implementing, and using EHR systems at Beta. The opportunity to triangulate between these data sources enhanced our understanding of the evolving process of implementation.

### Data analysis

Data analysis was an iterative process. We followed a two-step approach: initially, at Beta case study level using a combination of deductive and inductive approaches [33,34], and then a meta-synthesis that drew upon the analytical themes from other case studies, which were predominantly in acute hospitals. We used an adapted sociotechnical framework for data organiziation and classification of findings [27], and ‘sociotechnical changing perspective’ [35] for data analysis and interpretation, which we discuss below. Further, we presented primary findings from each case study through two complementary fora: regular analysis workshops with the wider evaluation team and formative feedback sessions with hospital representatives. This helped with validating the case study findings and furthermore integrating findings with our broader evaluation, enabling us to draw out some transferable findings.

### Our theoretical perspective: ‘sociotechnical changing’

Most EHR evaluations have drawn upon a broadly positivist ontology with a view to making causal inferences about the effectiveness of EHRs [30]. In the context of our evaluation, it was however not possible to ‘control’ for contextual factors using standard experimental or quazi-experimental designs. Moreover, it was also important for us to be cognizant of the fact that both the social and technical dimensions of EHR had the potential to shape each other over time in the complex and evolving environment of healthcare settings [36]. A number of theoretical frameworks have been developed and deployed to study this reciprocal relationship between EHR and the organization including: the role of leadership and envisioning the implementation of EHR as change management [37]; engaging with various groups of stakeholders [38], taking wider social context into consideration [39]; integration of EHR with workflows and care pathways [40]; organizational culture and behaviour [41]; and the ever-evolving contextual flux [42]. Drawing on these theoretical prepositions, Aarts et al. (2004) [43] has highlighted three dimensions to understanding the implementation of EHR: the interrelation of the organizational environment and the technology; a constantly changing mileu of the organization and environement: “emergent change”; and the interaction between system’s functioning, the organization’s needs and working patterns: the sociotechnical approach [44].

Our approach [30], pursued over the course of a 30 month longitudinal evaluation of national EHR systems in the English hospitals [21,24], underscored the emerging nature of change and its characteristics when evaluating EHRs. We refer to such change, as it happens as ‘changing’ (present participle) [35]. This implies that the EHR software, the clinical practice, the care giving, the organizational structures, and the carriers of institutional and professional norms were all in a state of flux, moving from somewhere now lost in the past to somewhere in the uncertain future: ‘becoming’ [45]. We therefore focused on the activity ‘in between’, the period of implementing EHR during which things were changing, rather than some predicted state of achieved change.

In our analysis, we adopted a social construction [46] and performative view [30]. We applied this performative view to explore how a diverse set of stakeholders [47] performed to make sense of new circumstances under EHR and make it work [42,48], enabled and constrained, as they were by their own skills, attitudes and the various technologies and other resources available to them. We sought to explain how stakeholders’ understandings and actions shaped adoption or non-adoption of RiO at Beta [49,50]. This is what we call the notion of “working-out” to signify a dynamic process of change over time that involved the ensemble of people, existing and emerging work practices and tools, individuals and organizational beliefs, assumptions, and expectations [48,51,52], which can be understood as both cause and consequence of longer-term processes of changing [53].

We sought to explore, understand and narrate the stories of EHR “in-the-making” [54,55]. Thus, we were less concerned with assessing the progress or achievements of implementing EHR systems measured against predefined criteria of success or failure, expectations and project milestones [43]; rather, drawing on the principles of Actor Network Theory (ANT) [54,56], and other studies on impact of perceptions of EHR systems on the implementation [e.g. [52]], our focus was on exploring what people understood about EHR (perceptions, hopes, fears) and what they actually did in their day to day practices (uses and practice) to ‘make it work’.

## Results

We report on four main findings. First, we describe the arrival of EHR at Beta through reflecting on the underlying reasons that led Beta to decide to implement RiO. Second, we provide an overview of the process of implementation through describing the management strategies pursued by Beta in implementing the software. Third, we consider the experiences, perceptions, and attitudes of users with RiO software. Finally, the local consequences, including some early benefits of the EHR software, realized by users at Beta will be described.

### The arrival of EHR at Beta

Compared to acute hospitals, many of which traditionally used some sort of computerized systems to manage or deliver patient care, mental health settings in England at the time of this study typically lacked any ‘joined up’ electronic information system. It has been suggested that there is ‘an intrinsic lack of interest in information systems among many staff in mental health’ [57]. It is therefore perhaps unsurprising that computerized patient administration system (PAS) consisting of basic patient demographics, with little or no clinical functionality, had hitherto been the dominant form of electronic records in mental health settings. The organization of mental health Trusts (the administrative unit in England, which can include one or more hospital or clinic) involves a close working relationship with primary care and social services to manage a range of often complex cases involving several stakeholders. Episodes of care in mental health hospitals typically last longer than in acute settings, on occasions up to several years. Record keeping is also very different from the approach used in the acute sectors as the notes tend in mental health settings to be more narrative in nature. Paper record systems were the standard method of record keeping in mental health settings, these offering the advantages of being self-contained, (manually) transferable between clinical locations and well suited to narrative-based recording of clinical entries [58]. Consultations also tend to last longer (about an hour) and consequently notes tend to be very long:

"“I suppose our note keeping is very different because it’s therapeutic so we’re writing an hour session where it’s just based on talking to somebody so our notes are a lot longer they have a lot more detail…” (Nurse)."

NPfIT initiated the introduction of integrated EHR software with clinical functionality to mental health hospitals. The financial benefits of the hospital being part of NPfIT, i.e. virtually free software and support for early adopter hospitals (up until 2015), as well as RiO’s ability to connect to the national Spine [1] led Beta to implement the EHR software procured by NPfIT:

"“You couldn’t really say how long it [the legacy PAS software] would develop or be in existence with the bigger systems coming in. We had to take a view then what is our strategy through to 2015 on this to get that developed” (Manager)."

Beta perceived the deployment of a modern clinically-oriented EHR as an essential step to maintain Foundation Hospital status (which would provide greater financial independence from the central NHS). EHR was seen as an opportunity to strengthen the information technology (IT) at Beta:

"“We’ve probably invested in the structure around this project [EHR] more than we’ve invested in anything else. We’ve employed a lot of external people to come in and roll this project out and quite a lot of investment in rolling it out. This is pivotal to improving our IT capability” (Doctor)."

Beta perceived the NPfIT-procured EHR as a potential enabler, facilitating integration of its care services with other care settings in their region and nationally. Moreover, as an ‘early adopter’ of NPfIT-supported EHR software, Beta was financially incentivized to implement RiO.

### The process of implementing EHR

Implementation of RiO system into Beta proved challenging, the key issues encountered are described below.

#### Management of the implementation (the process of changing)

Beta followed an incremental approach in implementing RiO. This was crucial in order to connect the main hospital and a number of community centres at Beta that were physically dispersed. RiO version 5.1, which was implemented in Beta, was the first version with connectivity to the Spine (NHS national database and messaging service). On the basis of various services and physical sites within the organization, Beta divided the implementation into three distinct phases. The hospital adopted a ‘big bang’ approach within each deployment phase, in which most services went live and were migrated to RiO simultaneously, over a single night. External stakeholders (interviewees who were not employed by Beta) stated that Beta managed to plan well and put reasonable infrastructure in place for implementing EHR; this had positive effects on the experience of EHR implementation:

"“I’m very impressed here [Beta]. They understand the importance of RiO and implementing it correctly and I was surprised to see the seriousness that they’ve taken the project and therefore, the amount of resources that are allocated to it. That is the reason that I’m confident that RiO will be implemented successfully and the Trust will benefit from it” (NPfIT)."

RiO was not however linked to the local authority databases specified for social care services. This proved to be a major barrier to the integrated provision of mental and social care services in England, and led to data entry duplications, which in turn had adverse effects on users’ attitude towards the EHR:

"“You’d like to sort of knock their heads together and say, yes, it’s wonderful having a [city name] wide solution for mental health. Why didn’t anybody think of integrating social services into it? This is the problem that existed for a long time” (Manager)."

Beta used virtual databases for training purposes, which were criticized for not being rooted in users’ needs or their actual work practices:

"“Training was not useful for what we needed to know to be able to do our jobs. It didn’t tie in our processes and PIs [performance indicators] and things like that” (Manager)."

This led to “*when users actually go back onto their desk they realize, oh, I can’t remember this, and because their drop downs are totally different.”* (Manager). Consequently, most interviewees preferred to learn through using the software in practice rather than in traditional classroom environments.

Beta mostly outsourced the implementation team responsible for putting RiO in practice with experienced people, who had deployed RiO in other settings. This was perceived to be a significant advantage:

"“I think key to our success here is that you get an individual in each of the work streams that has RiO knowledge and has gone through deployment and understands the problems” (IT Manager)."

Nevertheless, despite the advantages, there were two main disadvantages to this: high deployment costs; and challenges with employing a team predominantly comprising of temporary staff who were likely to be shed when returning to business as usual, with the potential for considerable loss of experience and expertise.

### Users’ experiences with EHR software

In this section, we describe the experiences of EHR users at Beta and the ways that various users ‘worked out’ what EHR was and how to use it in their day to day practices. To begin with, some interviewees wished that the software was designed more around clinicians’ needs: “*It would be interesting to know how many medical types were involved in the setting up of it”* (Nurse). RiO was described as “*too clumsy*” (Nurse), *“quite an old looking tool”* (Doctor), and not easy to navigate:

"“It is disappointing to have a clinical tool that is not as advanced as what I can do when I go and do my Internet shopping for my Tesco weekly shop” (Doctor)."

RiO was thus seen as being unfit for at least some clinical purposes. Some described RiO as being designed on a simplistic, linear interpretation of the workflows in mental health settings. They thus criticized the software as not reflecting the contextual differences across care settings:

"“The reality is completely different. We see lots of different people. The type of contact we have with people is completely complex and it’s very variable. It varies from hospital to hospital how people deal with their clients. We had to adjust to that” (Care Manager)."

Although RiO was an established software that had existed in the British market for more than a decade, it was seen as lacking some essential assessment and clinical functionalities:

"“In old age psychiatry we use a Single Assessment Process (SAP) which isn’t on RiO at all. We are just going to have to continue using that as a Word document and uploading it. The core assessment is completely unsuitable for our use” (Doctor)."

The degree of cultural change as a result of using EHR left some users feel uncomfortable:

"“It was quite a shock to not being able to do the things that we used to do on ward. For example, risk assessments used to be just typing little bits. We need to do everything in a different setting in RiO” (Nurse)."

On the one hand, some clinical functionalities of RiO including ICD (International Classification of Diseases) 10 coding for patients’ categorization were seen as *“not perfect, because there are a number of diagnosis that aren’t coded. That’s all we’ve got and you have to fit people into it”* (Doctor, Beta). This added to consultants’ workload because *“ICD coding has to be a consultant that puts it in and can’t even be a junior doctor, so that’s just slightly irritating to me”* (Doctor).

On the other hand, there were a few functionalities that users perceived as unnecessary and irritating:

"“You can’t delete out the bits that aren’t relevant, so you would have the whole document which includes things like forensic history and murder, which are perhaps not appropriate to an elderly person with some mild memory problems” (Doctor)."

Many interviewees perceived RiO as being incapable of meeting a number of important expectations of users. For instance, the very sensitive and distinct therapeutic nature of the relationship between patient and carer in mental health settings was not, it was suggested, appropriately considered by the designers of the RiO software. Given the very scattered distribution of community centres affiliated with mental health hospitals, it was perceived that the implementation team underestimated the practicalities of real-time data entry at the point of care:

"“I need a clinical tool that I can sit here and stick my card in and look at the patient notes from my patient’s that are up the road at a different hospital and enter notes and read what the community worker has done and in their conversation with the family” (Doctor)."

Several practical issues with regard to the day-to-day use of RiO arose. For example, frustrations were reported because of staff being automatically logged-off if the software was not used for 30 minute. In addition, RiO was used on desktops in an office environment where the public had no access, so it was seen as “secure enough” to ensure confidentiality – however, Smartcards (a chip and PIN card that grants access to patient information based on healthcare practitioners’ work and level of involvement in patient care) were sometimes left in computers.

Logging-in and -out of multiple systems (including the legacy systems and the dataset for social care services which ran separately from RiO) was viewed a time consuming, unnecessary and cumbersome procedure, which *“defeats the purpose”* (Manager).

Depending on their position and responsibilities, staff used the EHR applications differently. For instance, senior psychiatrists used RiO less than their junior colleagues:

"“I probably use it [RiO] less than 10% of the time. It would be my junior doctor that’s inputting the information, not me. They probably use it 80% of their day” (Doctor)."

Other staff groups at Beta who had to enter data complained about increased administrative burden and, as a result, a reduction in time they had available to spend with patients.

### The local consequences of EHR systems: early benefits of RiO at Beta

Despite challenges and difficulties, our evaluation revealed several perceptions of positive changes in work practices and patient care as a result of using EHR systems at Beta. These are described below.

#### Changing work environment

Generally, implementation of EHR systems brought the hospital the opportunity to strengthen its IT infrastructure:

"“RiO pushes IT to the front and just as important as clinical practices. Therefore, the Trust needs to have an on-going budget to be able to maintain their IT equipment and also look at advanced technologies that can go directly into RiO” (IT Manager)."

This enabled, some interviewees envisaged, the hospital to transform a number of its daily work dimensions towards more efficient services:

"“We are at the beginning of a big organizational change. People don’t have to travel backwards and forwards. They could just work outside of the office all the time. Therefore, you see more patients in a day. Your electronic record is up to date. It just goes on from there” (Manager)."

Data sharing was also perceived to be quicker, more transparent and secure, and timely through using RiO:

"“EHR brought in the standardization process in all the practices. That was very key. Because of the data warehouse on EHR, as long as the data is put into EHR, we have the ability to report on every single field” (IT Manager)."

In addition, RiO was seen as contributing to the standardization of “*the context and structure of the letters that were sent to a patient, a GP or a carer”* (IT Manager), which increased consistency of record keeping and enabled data extraction.

Patients’ care was also perceived to have become safer, for example, because of improved access to important information, and *“the communication [with clinical colleagues] has improved a lot. There is just the reduction of clinical risk, the fact that information is available to all the clinicians who are involved in someone’s care”* (Clinical Lead).

#### Users’ attitude about the consequences of EHR

The attitudes of clinical staff varied with regards to the consequences of EHR systems on their work practices. For instance:

"“The audio landscape in the office has changed. It’s not phones ringing and people talking. It’s the kind of sound of typing in the office, which is a little bit creepy” (Nurse)."

Within the early stages of implementing RiO, some users expressed their stress and anxiety, stating that they had less interaction with colleagues and spent more time than usual sitting in front of computers.

Interestingly, and in contrast to the experience with Smartcards noted above, most interviewees were confident that EHR provided a more confidential record of patient data than paper:

"“The system will track the person who is illegitimately looking at my record and figure out why they are entering it” (Nurse)."

#### Increasing quality of care and improving patient safety

As described earlier, although Beta did not have much flexibility for software configuration following the NPfIT chain of deployment, *“that’s outweighed by we get a standardized build and overall I think that has been a great benefit to us”* (Manager). Clinical users consistently praised the ability to see patients’ notes on RiO quickly, completely, in real-time and live across the whole hospital and affiliated community health centres:

"“I think a great benefit [of RiO] is being able to access records. What we’ll be able to do when we have RiO is access the notes of where they’ve been seen, wherever it was within the organization” (Doctor)."

This was seen as bringing direct benefits to the patients because:

"“Sometimes RiO makes things for our patients easier. Instead of waiting for me to write a referral form and then send in CPA^b^and then send in risk assessment and then wait for them to meet them up, they can go on RiO and have a look at the CPA. It’s actually speeding things up and it’s more reliable about information which is live” (Nurse)."

EHR systems were perceived as an enabler to keep mental health patients’ information safer than the previously used paper-based system, particularly when patients moved across care settings or got transferred:

"“I think it’s very easy for things to get missed when people are being transferred from one site to another. Another advantage is when you are looking at progress notes, you can filter them. If you are going to find something, you will be able to find it much more easily” (Doctor)."

EHR was also seen as making communications faster and more reliable because *“you are not faxing and you are not saying, that fax machine not working. I’ll email it to you”* (Nurse). Further, EHR was perceived to assist more careful and systematic monitoring and greater efficiency when utilizing resource across mental health hospitals and affiliated centres:

"“Users must outcome their appointments every two days after it’s been actually conducted. We know exactly if the patient was visited or if it was cancelled. In that sense, we can do it a lot of tracking and a lot of monitoring and better performance indicators” (IT Manager)."

The ability to check progress notes was seen by some as an opportunity to monitor staff activities and, if necessary, take remedial actions:

"“Because I have RiO I can actually go into the record myself and I can see what who has been discharged… I can see that that person from that team actually didn’t record it in their diary, so that’s why the report didn’t pick it up. Then I can flag it” (Manager)."

"Quality of care was also seen as being improved because the EHR “allowed us to look at our practice and make it more transparent. We have got so tight with doing everything correctly. We contact the GP and email the assessment out within three days. We want to make sure it’s done properly” (Care Manager)."

In addition, healthcare providers were less anxious about misunderstandings and mistakes about their planned orders for patients being carried out correctly:

"“When I’m on RiO, I’ll just quickly type in what I expect my nurse to do, at the time, rather than thinking, well, she’s got the notes so hopefully she’ll make the entry confirming what I’ve said” (Doctor)."

Also, clinicians found the availability of information valuable:

"“I obviously get a lot of phone calls from patients, involved professionals and carers. You maybe don’t remember the exact and you don’t have the notes, immediately to hand. Obviously, now I’m on the phone, I’ll be able to tap in and get the details up [on RiO] and make any changes or suggestions, I can immediately type them in as well” (Doctor)."

This partly happened because users became more aware of the need to write patients’ notes more accurately:

"“I think now, with RiO it makes it all the more visible and people have to be more careful about how they write things” (Manager)."

The greater visibility of health records led to reducing patients’ risk of poor treatment because of missing data or actions:

"“I have better quality of care now. There is nowhere to hide with EHR. If you didn’t put something down, it’s going to be missing and you can see straightaway” (Care Manger)."

Given the very text-based environment of mental health, EHR was perceived to have improved patient safety by enhancing readability of patient notes:

"“The main thing really is that we can read people’s writing. That was a big thing before that you couldn’t actually read what people were writing in the NHS across the board” (Nurse)."

This advantage was more visible when staff were on leave and their assigned patients were taken care of by other members of the team:

"“The ward might have not put in the community slant of things on their ward notes. And then information would have gone amiss or they would have been delayed. Now I can just log in and have a look at the patient notes and I can see what the ward has entered” (Nurse)."

"Further, “using [electronic] records for other purposes like research is much easier now” (Doctor, Beta)."

"All-in-all, the EHR was seen to have “played a key part here to push the hospital to be more modernized. Be more electronic orientated. I think that’s the most benefit that it brings” (IT Manager). As a result, “when we were going to negotiate or bid for contracts with the PCT (Primary Care Trust responsible for purchasing services from Beta, now being restructured), we had more accurate figures on which to base our bid. This very much helps the business function of the Trust” (Senior Manager)."

## Discussion

Our adapted ‘sociotechnical changing’ framework has three main dimensions: the constantly evolving nature of the contexts: i.e. environment, organization, perceptions, and consequences of the implementation of EHR systems; the performative nature of evaluating the implementation and adoption of EHR to explore how it ‘worked out’ and was ‘made to work’; and finally exploring and narrating the implementation of EHRs ‘in the making’, beyond the potentially misleading dichotomy of success or failure. Such an approach enabled us to learn how the EHR was formed, translated and reproduced in various entities at Beta [59] and the different meanings it embodied for various stakeholders, at different times and locations [60]. Our study revealed the usefulness of this approach to shed light on empirical aspects of the implementation and adoption and to plan for improving the process.

The decisions to procure EHR, the selection of the specific software (RiO), the process of implementation and the attempts to make RiO work at Beta, all proceeded in a rapidly changing NHS environment. NPfIT was dismantled in 2011. If Beta had decided not to join NPfIT, the organization may have lost the opportunity of being an ‘early adopter’ of NHS-centrally procured EHR systems. At the time of making the decision to proceed in 2008, the financial and non-fiscal incentives to be early adopter, and Beta’s desire to seize the opportunity of integrated EHR to get closer to the Foundation ^c^ status to help the organization survive, were constantly and quickly changing over time. For stakeholders at Beta, EHR embodied certain interests of (e.g. senior managers, doctors, IT staff, managers, etc.) that was linked to systems of politics and power relations [48], which shaped perceptions and actions, as opposed to a discrete and contextual resource deployed in planned processes of change [42,61]. In hindsight, irrespective of shortcomings of the implementation and some negative experiences by the users, Beta’s decision was a right choice for the organization and the quality of care for its patients.

Our performative and social construction view helped explore the implementation of RiO in the making and portray how users from various disciplines shifted their perceptions and attitudes towards the EHR system in use, and became generally positive to make it work for their organization. In this way, change is rarely a fast or direct movement from ‘the old’ to ‘the new’, rather the new is born within the old and co-exists with it, and the old and the older still remain sedimented within the most new [35,54]. In addition to capturing what people said they did versus what they were doing, we managed to reconcile between the state of being (e.g. being a nurse, or doctor, or computer, etc.) and practice of doing (e.g. order entry, putting notes, etc.) [35]. Our longitudinal evaluation allowed us to understand the implementation process through engaging with actors who experienced changing in their daily interaction with EHR, and who also were being changed by it.

Initially, the users expressed mixed feelings about RiO and perceived it as being somewhat inadequate. They complained that it lacked some key clinical functionality versus loads of useless functions on RiO, and the significant cultural and work environmental changes that EHR brought to mental health settings. For instance, in line with the literature, some clinical users were concerned about adverse effects of EHR on healthcare practitioner-patient relationship [14,15,62,63]. However, a lot of users’ initial anxiety, negative attitudes, and stress were replaced with hope and satisfaction. This partly happened as a result of attempts to make RiO work and appropriate preparation to adopt EHR, which led to experiencing and recognizing some early benefits. Modest early benefits led users change their behaviour in substantial ways, many of whom, including doctors and nurses mentioned the greater degree to which they paid attention to creating more accurate and meaningful notes on RiO, because they were constantly seen and judged by their colleagues.

Our theoretical perspective helped ensure that we did not reduce the EHR to delivery, implementation and immediate use [53], but understand it as both cause and consequence of longer-term processes of changing, during which people and EHR came together to perform actions and tasks [56] as co-constitutive entities [43,55]. Such a social and cultural shift did not happen serendipitously and over a night. Rather, we noticed that the vision of change management [64], the leadership of the organization who made the decision to join the NPfIT despite the negative climate and the uncertain future of NPfIT, and constant support and help from senior management who invested in appropriate infrastructure, were the main reasons behind changing towards improvement, and reducing degrees of resistance to adopt EHR, and making RiO work at Beta.

The process of the implementation of RiO, as we understood it, involved multiple intricately woven moments of *changing* including *inter alia* combinations of the organizational, technical, social, professional and care, which was materialized as it was performed by various stakeholders with different sets of attitudes and perceptions, at different times and locations, across our context of investigation: Beta. This led us to learn insights that could be obtained from approaches that sought to ‘tell the whole story’ not just the ending [30,65]. Our ‘sociotechnical changing’ framework enabled us to manifest changing by capturing stakeholders’ perceptions of EHR as instances of both *projection* (what is possibly becoming new) and *remembrance* (what is old and difficult to give up) [42]. For us, studying implementation and adoption of EHR was inevitably and eternally a process or performance, suspended between what was and what might one day be. The EHR thus comes into being as and when it is performed (not when software is delivered and installed) even to the extent that it ‘vanishes when it is no longer performed’ [66]. In addition, we observed redistribution of professional responsibility and degrees of job change as users attempted to inscribe their interests into EHR [50]. Initially, there were complaints about extra burden of administrative job. Some users, senior doctors in particular, for whom administrative job was conducted by junior doctors or nurses traditionally, were reluctant to put notes on RiO. Nurses, in contrast, were generally more compliant as they projected EHR as a chance to take more control over their work (remembrance).

Further, by exploring EHR system (RiO in this case) in-the-making, we focused on real concerns of policy makers and managers – the causal texture within which the implementation happened. Our findings brought to the fore the intricate set of interlocking changes in practice that EHR implies, a more formative view than the image of discrete change, and a detailed stock of knowledge that informed key stakeholders at the time that it was, we believe, most needed. For illustrative purposes, we refer to feedback, recognizing that our ‘sociotechnical changing’ approach resulted in useful outputs that informed strategies and brought improvements to the implementation of RiO at Beta:

"‘…excellent stuff that truly gave us insights we as a deployment team had not perhaps fully thought about or understood… I think a second phase review of perceptions of the system after it has settled down would be extremely beneficial, warts and all, and help with the formation of our future strategies and approach.’ (Senior manager)."

Although not optimal, people worked hard around issues to make the system more compatible with their organizational needs. As a result, they harvested some modest benefits for both patients and the organization [67] and valued the system [37] eventually. From our perspective, non- or partial adoption but also rejection, mis-use, non-use, resistance to EHR and workarounds, all are not simply negative effects, pathologies or signs of failure, but are alternative enactments upon technology, which may pave the trajectory of organizational learning towards future smoother implementation process [42,68,69]. In this way, as an intertwined product of technology; work practices; and people who make them work, EHR is made to actively produce a fit system to the needs of organization [43].

All in all, the ‘sociotechnical changing’ perspective helped us move away from static before and after implementation ‘impacts’ or notions of discrete change. Instead, we focused on nominalism (rather than essentialism), crossing of temporalities (rather than before-after dualisms) and practice (rather than strategic or functional) orientation [35].

Our findings are in contrast to the claims that EHR may lead to impersonal and inaccurate clinical notes in mental health settings [11]. Given a great desire of mental health patients to receive a copy of their summary notes (78% of patients reported that it was helpful to receive the letter, and 83% reported that they would like to continue receiving them) [70], EHR may lead to enhanced patient satisfaction by producing more accurate notes. The evaluation of the implementation of EHR in an NHS community mental health setting showed similar results: high degree of users’ satisfaction and some tangible benefits to clinical staff [71].

### Lessons for implementers

On the basis of challenges encountered during the implementation of EHRs, and early benefits realized at Beta, we consider some policy implications below that may help facilitate the improved implementation of EHR systems into mental health settings.

First, stakeholders need to be identified prior to planning to procure and implement EHR software, and their computer literacy and ability to access the technology needs to be adjusted accordingly [72]. Engaging with healthcare professionals from early stages of planning and as EHR partners is pivotal to maximize efficacy and improve patient care.

Second, although overlooked by the NPfIT, it is important to understand whether both mental health service providers and users would like to have EHR systems–and for what purposes–before embarking on the large-scale implementation of EHR systems.

Third, EHR needs to be seen as a sociotechnical entity by stakeholders, thereby ensuring a user-centred design of EHR [73,74]. It is important to address concern of users who may present less interest and enthusiasm about EHR.

Fourth, contextualization and taking heterogeneity across mental health settings is crucial to implement EHR initiatives. This might also help identify areas in need of additional support when implementing EHR software.

Fifth, given a huge cultural shift that EHR brings [75] to heavily text-based notes in mental health, healthcare practitioners must be educated and protected with regards to transparency and observing confidentiality of patient notes.

Last but not least, the safety of EHR systems needs to be ensured prior to and during the implementation [76], and their efficacy requires to be evaluated using robust, independent, and forethought evaluation programs that employ reflexive and multidisciplinary research team [30].

### Strengths and limitations of this work

Our findings need to be interpreted with caution. We evaluated one ‘early adopter’ mental hospital in England, during a relatively short period of EHR implementation and in the beginning of a long journey to full integration. We did not intend to evaluate the software specifications *per se*. Rather, we attempted to understand what was ‘going on’ in terms of the implementation and adoption of EHR in the studied settings, namely the process of implementation not outcomes. The in-depth case study approach [28,30] was helpful to ensure an understanding of the contextual aspects of the implementation, however generalizable lessons can only be drawn with great caution. In addition, our adapted ‘sociotechnical changing’ perspective may have narrowed down our focus on the micro level, thus hindering the bigger picture to be portrayed. Nonetheless, we managed to collect data from various stakeholders from outside Beta, and compared our analytic themes with other case studies in our evaluation. This may have expanded our understanding of the phenomenon. We acknowledge that many perceptions and attitudes described here may be altered in times to come, as there is a natural learning and adoption curve in any organizational change initiative. Finally, we did not study patients in our evaluation. Other studies on the impact of EHR use on the quality of the patient-psychiatrist relationship found no change in satisfaction scores among adult psychiatric patients for whom EHR was used during outpatient encounters instead of paper charting [7].

Nevertheless, despite the above limitations, little has previously been published on EHRs in mental health settings, let alone in the context of national implementation endeavors. This paper may shed light on some practical dimensions of EHR implementation and things to consider when planning implementing integrated EHR systems in mental health settings. As such, we hope that will help in the many future implementation and adoption of EHR systems in mental health settings that are now underway or are planned in countries with similar healthcare systems, and possibly beyond.

## Conclusions

There is now a strong policy drive to implement EHRs in mental health settings. Despite substantial initial challenges, the English mental health hospital reported on in this paper achieved some early-perceived benefits from implementing the EHR system. These benefits related to improved legibility and accessibility of patient records, and transparency of care processes. Because of the nature of mental health and the specific conditions of its patients, some of them have difficulty in describing their problems and occasionally their medical history appropriately, shared electronic records proved to be potentially useful for their safety. As mental health settings face greater challenges for providing a quality service at an acceptable cost, wise implementation of suitable EHR applications may boost the chances for the success.

## Endnotes

^a^ A collection of national applications, services and directories that support the NHS and the exchange of information across national and local NHS systems. The project began in 2003, and now every NHS organization can access the Spine services (http://www.connectingforhealth.nhs.uk/systemsandservices/spine).

^b^ Care programme approach: Anyone experiencing mental health problems is entitled to an assessment of their needs with a mental healthcare professional, and to have a care plan that is regularly reviewed by that professional (NHS Choice 2012).

^c^ Greater autonomy and freedoms for NHS hospitals within a national framework of standards [DH 2005].

## Competing interests

The authors declare that they have no competing interests.

## Authors’ contributions

AT undertook data collection and analysis and drafted the first version of the manuscript with AS and NB, who all extensively contributed to several revisions and intellectual development of the article. All authors read and approved the final manuscript.

## Pre-publication history

The pre-publication history for this paper can be accessed here:

http://www.biomedcentral.com/1472-6963/12/484/prepub

## Supplementary Material

Additional file 1 Interview topic guide: NPfIT & external stakeholders. Click here for file

Additional file 2 Interview topic guide: Healthcare professionals and managers. Click here for file

Additional file 3 Interview topic guide: Implementation Teams. Click here for file
