# Supplementary material for: We are bitter, but we are better off: case study of the implementation of an electronic health record system into a mental health hospital in England
Source: BMC Health Serv Res. 2012 Dec 31;12:484. doi: 10.1186/1472-6963-12-484 (PMC3545968; doi:10.1186/1472-6963-12-484)
Supplement: Additional file 1 — Interview topic guide: NPfIT & external stakeholders. [file 1472-6963-12-484-S1.doc]

Appendix 1: Interview topic guide: NPfIT & external stakeholders

**Interviewee’s Background**

Job role

Length in service

**Implementation**

Challenges faced concerning the development and implementation of EHR software

Methodology followed for EHR software development

Testing process: steps, problems reported

Process of addressing issues that Early Adopter sites raise

Strengths and weaknesses of EHR software

Resources NPfIT has dedicated to early adopter sites

*Software outsourcing*

**Perceptions**

Role of NPfIT/LSP//Software houses/SHA in the Programme

Achievements from the adoption of EHR software in early adopter sites

Issues/difficulties they faced from the adoption of EHR software in early adopter sites

Collaboration and communication process between different stakeholders (SHA, NPfIT, Trusts)

Consequences of the political and economic context on the NPfIT and EHR

Contract: issues and obstacles

Lessons that can be transferred to future implementation sites/practices

Evolution of EHR in the future

Standardisation and/or localisation of the implementation process: views, rationale, benefits and disbenefits.
