# Supplementary material for: We are bitter, but we are better off: case study of the implementation of an electronic health record system into a mental health hospital in England
Source: BMC Health Serv Res. 2012 Dec 31;12:484. doi: 10.1186/1472-6963-12-484 (PMC3545968; doi:10.1186/1472-6963-12-484)
Supplement: Additional file 2 — Interview topic guide: Healthcare professionals and managers. [file 1472-6963-12-484-S2.doc]

Appendix 2: Interview topic guide: Healthcare professionals and managers

#### Interview Guide for Healthcare Professionals (and other users of the systems)

Note that sections in italic are common with section in Implementation Team Interview Guide.

***Interviewee’s Background:***

- Current position in the organisation
- Relation to EHR

***Background about the current status of EHR****:*

- Software
- Release
- Functionality being used & future upgrades
- Location of use and users (ward, clinics, departments etc)
- Previous systems that EHR software replaced and other current systems
- What systems did you have prior to EHR? What for?
- Are there any systems in place for patient management like vital sign monitoring; or is there going to be?
- What is the level of integration of existing systems, e,g together and with EHR

**[Some users – mostly the super users – have been involved in the implementation process. In this case, we also use the questions from the Implementation section**]

**Use of NHS EHR software**:

- Previous systems that NHS EHR software replaced
- How the interviewee uses the system
- Changes in the way you use the system
- Training received and ongoing support
- IT literacy and skills – your own – your team etc.
- Tasks carried out through the system
- Frequency of use/ conditions of use
- Initial, current and ongoing problems and concerns
- Changes that the user would like to see happening in the system
- Role-based access & access to the Spine

**Changes that the system has brought about**:

- New tasks that have been added
- Old tasks that have been eliminated
- Same tasks done in a different ways
- Workarounds
- Modes of collaboration with other healthcare professionals
- Modes of interaction with patients
- Preparation of (new) standard operating procedures

***Consequences of the NHS EHR on:***

- Quality of Healthcare
- For Patients & patient pathways
- Healthcare professionals
- Trust
- Local Community
- Connection to and collaboration with GPs and PCTs
- Changes in your expectations

***Perceptions***

- NHS EHR in the future (local and national level)
- What would you do differently?
- Is it necessary?
- Is it worth it
- Benefits that you realised so far
- What is it all about?

Is the NHS EHR an end or a means for other changes
