# Supplementary material for: We are bitter, but we are better off: case study of the implementation of an electronic health record system into a mental health hospital in England
Source: BMC Health Serv Res. 2012 Dec 31;12:484. doi: 10.1186/1472-6963-12-484 (PMC3545968; doi:10.1186/1472-6963-12-484)
Supplement: Additional file 3 — Interview topic guide: Implementation Teams. [file 1472-6963-12-484-S3.doc]

Appendix 3: Interview topic guide: Implementation Teams

#### Interview Guide for Members of the Implementation Team

**Interviewee’s Background**:

- Current position in the organisation
- Relation to NHS EHR

**Background to the current status of the NHS EHR**:

- Software
- Release
- Functionality being used & future upgrades
- Location of use and users (ward, clinics, departments etc)
- Previous systems that NHS EHR software replaced and other current systems
  - What systems did you have prior to NHS EHR? What for?
  - Are there any systems in place for patient management, like vital sign monitoring; or is there going to be?
  - What is the level of integration of existing systems, together and with NHS EHR

**Implementation/Adoption**:

- Decisions that were made (Who? What criteria?)
  - What were the reasons behind NHS EHR/moving to Rio
  - The way the business case was prepared; who participated, how approved? And changes to that?
- Who involved in implementation (groups and people)
  - IT literacy
- How
  - Steps that were followed
  - Methodology
    - Factors that influenced the implementation process (e.g. history, delays)
    - Changes in the implementation strategy
    - Issues of local configuration
- When (timeline)
- Incentives offered or given
- Resources used(human resources, financial)
- Changes in resources
- Training provided and ongoing support
- The method for training, real data or virtual – right software version? Was any material provided? Who provided, What form?
- What is the Trust’s strategy for new staff who need to use NHS EHR? Training, induction, SmartCard, etc.
- Management of data.
- Where are data kept and how are they managed?
- Collaboration within the organisation and across organisations:
- Software developer- NPfIT- Trust:
- Interests (differences and similarities)
- Mechanisms to encourage collaboration; how do you work together?
- Issue management process (who, how, what problems, mechanisms to resolve problems, examples of issues)
- Teething, current and ongoing problems
- What might be done differently?
- Awareness and Views about the contract
- Changes in the level of involvement of each organisation
- Early Adopters
- Feelings for being early adopter
- Mechanisms to facilitate collaboration among early adopter
- Lessons learned as used as input; as provided as output
- What can & cannot be learned & why?)

**Consequences of the NHS EHR on**:

- Quality of Healthcare
- For Patients & patient pathways
- Healthcare professionals
- Trust (management, strategy)
- Local Community
  - Connection to and collaboration with health economy (GPs and PCTs)
- Changes in your expectations

**Perceptions**

- NHS EHR in the future (local and national level)
- What would you do differently?
- Is it necessary?
- Is it worth it?
- Benefits realised so far
- What is it all about?
- Is NHS EHR an end or a means for other changes?
